# Synergistic effects of blood flow restriction training and beetroot juice supplementation on knee extensor strength and fatigue resistance in college athletes

**DOI:** 10.5114/biolsport.2025.150043

**Published:** 2025-05-14

**Authors:** Xudong Yang, Yue Lu, Hongqi Xu, Qing Liu, Dae Hyun Yun, Young Jin Moon, Helong Quan, Sang Ki Lee

**Affiliations:** 1 Department of Sport Science, Chungnam National University, Daejeon, the Republic of Korea; 2 Research Center of Exercise Capacity Assessment and Promotion, School of Physical Education, Northeast Normal University, Changchun, Jilin, China; 3 Jilin Green Food Engineering Research Institute, Changchun, Jilin, China

**Keywords:** Blood flow restriction, Beetroot juice, Nitrate, Muscle strength, Anaerobic power, Fatigue resistance

## Abstract

Combining beetroot juice (BRJ) supplementation with blood flow restriction (BFR) training shows potential to meet the dual demand for enhanced muscle strength and improved fatigue resistance. This study involved 21 male college athletes who were randomized to a BFR group (n = 10) and a BFR-supplemented BRJ group (n = 11, nitrate 8 mmol/day) for 4 weeks of isokinetic BFR training. The strength (at 60°/s, 180°/s, and maximal voluntary isometric contraction) and fatigue resistance of the knee extensors and flexors were assessed pre- and post-intervention using an isokinetic dynamometer and a 30-second anaerobic power test, respectively. The four-week BFR and BFR+BRJ interventions significantly (P < 0.05) improved knee extensor/flexor peak torque and power (at 60°/s and 180°/s), while also delaying the decline in knee extensor torque during the 100-repetition maximal voluntary contraction test. The BFR+BRJ group showed a more significant advantage in the second half of the contraction (51–100 repetitions; P < 0.01). In addition, both interventions significantly reduced the rate of decline in peak torque, peak power, and average power at 60°/s and 180°/s after fatigue, with the reductions being more pronounced in the BFR+BRJ group (P < 0.01). In the anaerobic power test, the BFR+BRJ group maintained a higher power output for a longer duration (P < 0.05) compared to before the intervention, whereas the improvement in the BFR group was limited to the initial 3–10 seconds (P < 0.05). The results suggest that BFR combined with BRJ is an effective training method for improving knee extensor strength performance and fatigue resistance.

## INTRODUCTION

The strength of lower limb muscles, especially the knee extensors and flexors, is essential for sports performance and injury prevention. The quadriceps, being the principal extensors of the knee, are crucial for lower limb activities such as running, jumping, and walking, and are vital for stabilizing the knee joint during dynamic activities [1]. Inadequate muscle strength or fatigue resistance may result in poor joint mechanics and increased risks of anterior cruciate ligament tears, patellofemoral pain syndrome, and knee osteoarthritis [2]. Knee extensor strength is closely correlated with explosive performance in athletes participating in sports that necessitate repetitive, high-intensity leg motions, such as soccer, basketball, and track and field. Impairment of the quadriceps due to weakness or early fatigue diminishes performance in these motions [3, 4]. Thus, enhancing knee extensor strength and fatigue resistance has become a central focus in sports science, rehabilitation, and injury prevention.

In recent years, blood flow restriction (BFR) training has attracted attention for its effects on muscle hypertrophy and strength in athletes, rehabilitation populations, and persons with chronic illnesses. BFR training employs cuffs to partially restrict venous return and arterial blood flow [5], resulting in a localized hypoxic environment in active muscles. This condition heightens metabolic stress and enhances the recruitment of fast-twitch muscle fibers [6]. This environment promotes metabolite accumulation in skeletal muscle, which enhances cardiovascular adaptations [7], hormonal responses [8], and muscle oxidative capacity [9], while stimulating protein synthesis [10] and myogenic stem cell pathways [11], thereby improving muscle fiber structure and function even at low loads. Recent studies have verified that BFR training significantly enhances muscle strength and hypertrophy in collegiate and semi-professional athletes, positively impacting athletic performance and fatigue resistance [12].

The integration of BFR with isokinetic training has been investigated as a potent method to augment knee extensor strength. Isokinetic training enables precise assessment and improvement of specific muscle groups by controlling the speed of muscle contractions [13]. Isokinetic training, when combined with BFR, can enhance muscular adaptations. Studies demonstrate that the incorporation of isokinetic concentric/eccentric training with BFR significantly improves peak torque in knee extensors and flexors and effectively reduces the decrease in knee extensor torque after fatigue from running, thus preventing sports-related knee injuries [14]. However, impact of integrating BFR with isokinetic training in both professional and amateur athletes need additional investigation. This study aims to provide insight into this research area.

Beetroot juice (BRJ) has attracted interest for its potential to enhance athletic performance, delay fatigue, and facilitate recovery. It contains high levels of betaine [15] and nitrates [16]. Betaine possesses antioxidant and anti-inflammatory characteristics [17], efficiently neutralizing reactive oxygen species (ROS) generated during strenuous exercise, thus alleviating the inflammatory response associated with muscle fiber damage and accelerating recovery [18]. The nitrate concentration in BRJ is transformed into nitric oxide (NO) by the nitrate-nitrite-NO pathway. This procedure facilitates vasodilation and improves the delivery of oxygen, hormones, and nutrients to active muscles. Furthermore, NO enhances muscular oxygen usage efficiency and mitochondrial function, thereby facilitating enhanced muscular performance and recovery [19]. Recent studies indicate that dietary nitrate supplementation, such as BRJ, promotes muscular contractility [18] and improves fatigue resistance [16]. Notably, chronic BRJ supplementation has been shown to improve neuromuscular function by enhancing Ca^2+^ release from the sarcoplasmic reticulum [20], increasing muscle contractility in type II fibers, and optimizing excitation-contraction coupling [21]. The study by Daab et al. showed that chronic BRJ supplementation (containing 8 mmol/L nitrate for 7 days) significantly attenuated neuromuscular fatigue during a simulated football match [22]. Compared to the control group, BRJ supplementation significantly attenuated the decline in maximal voluntary contraction and reduced both central and peripheral fatigue. These findings suggest that chronic BRJ supplementation may contribute to improvements in neuromuscular performance and attenuation of the development of neuromuscular fatigue. In addition, long-term supplementation yields more pronounced outcomes in strength and fatigue resistance training [23].

While the mechanisms of BFR training and BRJ supplementation in augmenting muscle strength and fatigue resistance are distinct, the localized hypoxic effects of BFR, coupled with nitrate-induced enhancements in blood flow and muscle nitrate reserves, may result in greater significant metabolic effects in skeletal muscle. Studies indicate that low-oxygen environments enhance the conversion of nitrite to NO, highlighting the potential of BFR training to leverage the nitrate reserves stored in skeletal muscle [24, 25]. Although several studies have confirmed the individual effects of BFR training and nitrate supplementation, there is a lack of research and evidence on their combined benefits. The purpose of this study was to determine if the BFR paired with BRJ supplementation is superior to BFR training alone in terms of enhancing knee flexor/extensor strength, alleviating muscle fatigue, and improving exercise performance.

## MATERIALS AND METHODS

### Subjects

Twenty-four male undergraduates from the School of Physical Education volunteered for this study. The participants had consistent training experience in basketball, soccer, sprinting, or middledistance running. All participants reported no chronic illnesses, nitrate supplement use in the past six months, or recent injuries or surgeries. They were required to avoid mouthwash, sports supplements (creatine, β-alanine, arginine, citrulline and antioxidants), alcohol, and caffeine throughout the study. Written informed consent was obtained from all participants after explaining the study details. This study adhered to the Declaration of Helsinki and received approval from the Research Ethics Committee of Northeast Normal University (Approval ID: 20242026). Power analysis using G*Power 3.1 (Heinrich-Heine-Universität Düsseldorf, Düsseldorf, Germany) for repeated measures ANOVA (within–between interaction) determined a required sample size of 20 (α = 0.05, power = 0.9, Effect size = 0.4). A total sample size of 20 was required to achieve an actual power of 0.92.

### Experimental Design and Procedures

**FIG. 1:**
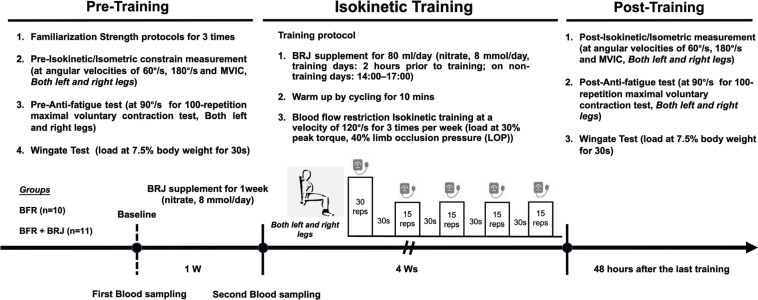
Schematic representation of the study design.

The study included a one-week BRJ pre-supplementation phase and a four-week bilateral knee flexor and extensor BFR training (3 sessions per week). Participants were stratified by body weight in 5 kg increments starting from a 65 kg baseline, and were randomly assigned to either BFR group (n = 12) or BFR+BRJ group (n = 12) using a block size of six to ensure balanced allocation. Three participants withdrew due to scheduling conflicts, resulting in 10 participants in the BFR group and 11 in the BFR+BRJ group. They completed three pre-intervention visits to familiarize themselves with the equipment. Based on current research recommendations for optimal nitrate supplementation [26, 27], the BFR+BRJ group consumed 80 ml of BRJ daily during the pre-supplementation and training phases (nitrate 8 mmol/day, M-ACTION, Shanghai, China), while the BFR group received an identical placebo (PLA). To mimic the color and earthiness of BRJ, PLA used food coloring and traces of cocoa powder. These were mixed with commercially available purple potato juice, which have a similar calorie content (217 kJ), protein content (1.5 g), and carbohydrate content (11 g) to the BRJ sports drink. To ensure effective double-blinding, BRJ and PLA are dispensed in opaque, unlabeled glass containers. Blood samples were collected twice in the morning at the same time, before and after pre-supplementation, to measure plasma nitrite levels. Before starting the formal training, baseline strength testing of knee extensors and flexors and a 30-second Wingate test were performed using the CON-TREX multi-joint isokinetic system (CON-TREX® MJ, Physiomed Elektromedizin AG, Schnaittach, Germany) and Monark ergometer (Ergometer 894E, Monark Exercise AB, Vansbro, Sweden), respectively [28]. These tests were repeated after the four-week intervention to evaluate changes in knee extensor strength, power, and fatigue resistance. The detailed experimental protocol is shown in Figure 1.

### Blood Collection and Analysis

Blood samples (10 ml) were collected twice in the morning in anticoagulant-treated tubes, centrifuged at 4°C (2000 × g, 10 min), and analyzed for plasma nitrite using a Nitrite Colorimetric Assay Kit (E-BC-K070-S, Elabscience, Wuhan, China).

### Isokinetic Training Protocol

During the four-week training phase, BFR and BFR+BRJ groups performed isokinetic BFR training thrice weekly. Training speed was set at 120°/s with resistance at 30% of peak flexion and extension torque (peak torque tested at each session using the CON-TREX system). Each session included 5 sets (1 × 30 and 4 × 15 reps) with 30-second rest intervals. Blood Flow Restriction cuffs (Thera tools Cuff 110 × 13 cm, Zhengzhou, China) were applied at 40% limb occlusion pressure (LOP), calculated as Thigh Occlusion Pressure (mmHg) = 5.893 (Thigh circumference) + 0.734 Diastolic Blood Pressure (DBP) + 0.912 Systolic Blood Pressure (SBP) − 220.046, based on Jeremy P et al. [29] and validated by Iván et al. [30]. The protocol is based on and adapted from previous studies and has been applied in several studies and has shown favorable results [31, 32]. Except for the three participants who withdrew for various reasons, all completed the training sessions three times a week for the entire intervention period.

### Maximum Strength and Fatigue Test Protocols

Before testing, participants completed a 10-minute warm-up on a stationary bike followed by lower limb stretching. Testing used the CON-TREX system with a spine-femur angle set to 110°, and motion range customized to each participant’s flexibility (0°-90°, where 0° corresponds to full knee extension). Following the 100 maximal dynamic knee extensions test protocol to induce fatigue and assess fatigue resistance [33]. Peak torque and peak power were measured twice before and after fatigue at angular velocities of 60°/s and 180°/s (5 repetitions each), along with maximum voluntary isometric contraction (MVIC) at a fixed 75° (5 s hold). The peak torque was defined as the maximal knee flexors and extensors moment (N · m) generated during concentric contraction, peak power as the highest instantaneous mechanical power output (W) calculated by multiplying peak torque with angular velocity (calculated by CON-TREX system), and average power as the mean value over all five repetitions. For the 100-repetition test, knee extensions were performed at angular velocities of 90°/s with maximal effort in extension (90°-0°) and assisted flexion (0°-90°) [34]. All participants completed the test on both legs, with the dominant leg defined as the kicking leg. The testing procedure was conducted by two operators, who were unknown to the subject, and who independently completed the subject’s testing.

### WinGate Test protocol

The BFR and BFR+BRJ groups completed two 30-second all-out Wingate tests on a Monark ergometer pre- and post-intervention. The load was set to 7.5% of body weight, and resistance was released automatically upon reaching 100 rpm [35]. Power metrics such as peak power, average power, power decrement percentage, and total work were recorded using Monark Anaerobic Test Software (COSMED, Italy). Power decrement was calculated as the percentage difference between peak power and power over time.

### Statistical Analysis

The data were analyzed using GraphPad Prism 9 software (GraphPad Software, San Diego, CA, USA). All data are presented as mean ± standard deviation (SD). Normality and homogeneity of distribution variances by Shapiro-Wilk and Levene tests of dependent variables. An independent samples t-test was used to compare descriptive characteristics between two groups. Plasma nitrite and muscle performance parameters (peak torque, peak power, time to peak torque, average power in 60°/s and 180°/s maximum strength test, and different ranges of average torque in 90°/s 100-repetition test) were analyzed using a two-way repeated measures ANOVA with group (BFR vs. BFR+BRJ) and time (pre vs. post) as independent factors. The decrease in peak torque, peak power, and average power over time during fatigue testing was also analyzed using this method. A three-way repeated measures ANOVA (group × fatigue × time) was used for knee extensor performance under fatigued and non-fatigued conditions, and the assumption of sphericity was assessed by Mauchly’s test, with Greenhouse-Geisser correction for degrees of freedom if violated. The effect sizes of the main or interaction effects were evaluated using partial η^2^ (partial eta-squared, pη^2^), where pη^2^ ≥ 0.01, 0.06, and 0.14 indicate small, medium, and large effects, respectively. Bonferroni’s multiple comparison test was used for post hoc analysis when main or interaction effects were significant, and the statistical significance threshold was set at P < 0.05.

## RESULTS

### Descriptive characteristics and comparison of variables between BFR and BFR+BRJ groups

There was no significant difference in age, height, weight, BMI, resting DBP and SBP, left/right Thigh Circumference, and Plasma Nitrite Concentration between subjects in BFR and BFR+BRJ groups before the start of intervention. Whereas, after one week of BRJ supplementation, Plasma Nitrite Concentration was significantly increased in BFR+BRJ group (Table 1).

**TABLE 1:** Descriptive Characteristics and Comparison of Variables Between BFR and BFR+BRJ Groups

Variables	BFR (n = 10)	BFR+BRJ (n = 11)	P-value	95% Confidence Interval (CI)
Age (years)	21.9 ± 1.58	21.63 ± 1.43	0.707	[-1.709, 1.118]
Height (m)	1.79 ± 0.04	1.78 ± 0.05	0.409	[-0.063, 0.027]
Weight (kg)	76.6 ± 5.71	76.09 ± 6.73	0.862	[-6.536, 5.518]
BMI (kg · m^−2^)	23.67 ± 1.17	24.02 ± 2.03	0.653	[-1.258, 1.961]
DBP (mmHg)	71.90 ± 4.74	73.27 ± 6.28	0.599	[-4.013, 6.759]
SBP (mmHg)	118.3 ± 6.48	120.45 ± 6.26	0.470	[-3.965, 8.274]
Left Thigh Circumference (cm)	55.4 ± 2.87	55.91 ± 2.26	0.688	[-2.106, 3.124]
Right Thigh Circumference (cm)	56.40 ± 2.94	56.82 ± 2.12	0.725	[-2.029, 2.865]
Pre-Plasma Nitrite Concentration (µM)	0.423 ± 0.052	0.445 ± 0.061^*^	< 0.001	[-0.358, -0.105]
Post-Plasma Nitrite Concentration (µM)	0.441 ± 0.053^#^	0.679 ± 0.166	< 0.001	[-0.374, -0.098]

Data are shown as mean ± SD. Abbreviations: BMI, body mass index; DBP, diastolic blood pressure, SBP, systolic blood pressure. Statistical significance is indicated as follows:

* indicates significant difference between post-BFR+BRJ and pre-BFR+BRJ;

# indicates significant difference between post-BFR and post-BFR+BRJ.

### Peak torque, time to peak torque, peak power, and average power for knee extensors and flexors at various test conditions pre- and post-intervention

**FIG. 2:**
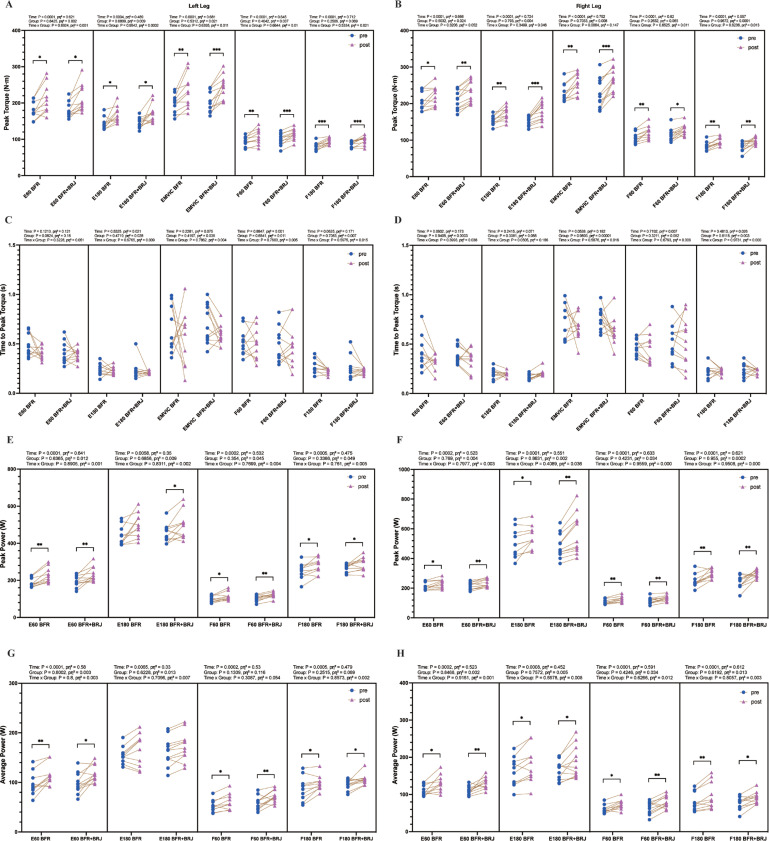
Pre- and Post-Intervention Comparison of Peak Torque, Time to Peak Torque, Peak Power, and Average Power for Knee Extensors and Flexors under Various Testing Conditions. Note: E60, extensors at 60°/s; E180, extensors at 180°/s; EMVIC, extensors maximum voluntary isometric contraction; F60, flexors at 60°/s; F180, flexors at 180°/s. Values are presented as mean ± standard deviation (SD). Statistical analysis was conducted using a two-way repeated-measures ANOVA (group × time) with Bonferroni’s multiple comparisons test for post hoc analysis. Main effects for time (pre vs. post) were statistically significant, while no significant interaction effects (group and group × time) were detected. Significant differences between groups indicated by * P < 0.05, ** P < 0.01 and *** P < 0.001.

The changes in peak torque, time to peak torque, peak power, and average power for knee extensors and flexors after the four-week intervention are shown in Figure 2. Results in Figure 2A and B indicate that the four-week BFR and BFR+BRJ interventions significantly increased the peak torque of knee extensors in both the left and right legs at 60°/s (P < 0.05), 180°/s (P < 0.05) and MVIC (P < 0.01). Additionally, both interventions significantly improved the peak torque of knee flexors in the left and right legs at 60°/s (P < 0.01) and 180°/s (P < 0.01). This may be due to the relatively low involvement of knee flexors in daily activities, resulting in a lower baseline strength. Consequently, flexors are more responsive to targeted training, achieving greater strength gains. No significant changes were observed in the time to peak torque for knee extensors or flexors in either leg at 60°/s, 180°/s, or MVIC testing conditions following both interventions (Figure 2C, D). The peak power of the knee extensors and flexors of both legs was significantly increased at 60°/s (P < 0.05) and 180°/s (P < 0.05) in the BFR+BRJ groups (Figure 2 E, F). Both groups exhibited a significant increase in average power of knee extensors and flexors in both the left and right legs at 60°/s (P < 0.05, Figure 2G, H). In addition, the average power of the knee flexors of both the right and left legs increased significantly at 180°/s in the BFR and BFR+BRJ groups, whereas only the extensors of the right leg increased significantly at 180°/s (P < 0.05, Figure 2H). The results suggest that BFR and BFR combined with BRJ interventions have similar effects on improving knee flexor and extensor strength.

### Isokinetic test of knee extensor strength at 90°/s over 100 repetitions

**FIG. 3:**
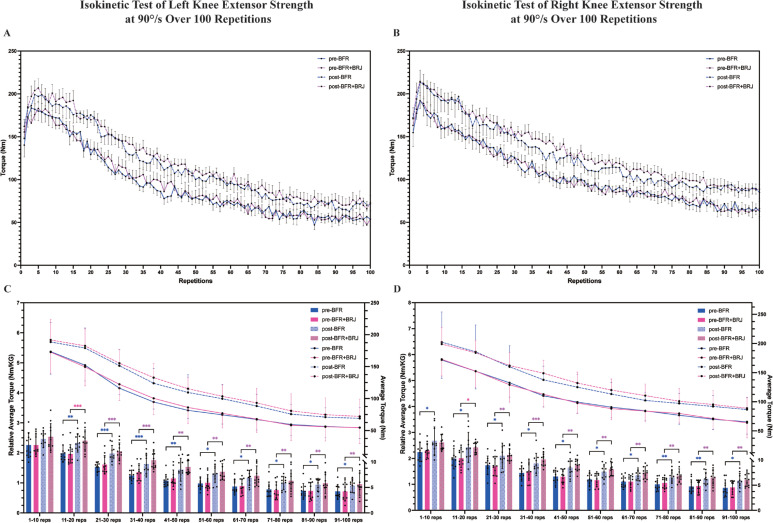
Comparison of Pre- and Post-Intervention Effects of BFR and BFR+BRJ on 100-Repetition Isometric Knee Extensor Maximal Voluntary Contraction Test. Note: Torque values of knee extensors over 100 repetitions (A, B), and the relative average torque and average torque (C, D) in different ranges (1-10, 11-20, etc.), pre- and post-intervention in the BFR and BFR + BRJ groups. Values are presented as mean ± standard deviation (SD). Significant differences between pre- and post-intervention are indicated by # for the BFR group and * for the BFR + BRJ group (* P < 0.05, ** P < 0.01 and *** P < 0.001).

Torque values of left and right knee extensors were continuously recorded during the 100-repetition test (Figure 3A, B). The results show that knee extensor strength in both the left and right legs increased after the four-week intervention in both the BFR and BFR+BRJ groups. To assess the effect of the four-week intervention on knee extensor fatigue resistance, the 100 repetitions were divided into 10 intervals for trend analysis of the average torque and relative average torque decline (Figure 3C, D). Results indicate that both BFR and BFR+BRJ interventions significantly increased left and right knee extensor torque in the 11–100 repetition range (P < 0.05). Notably, while BFR significantly improved the relative average torque of the right leg in the range of 0–10 repetitions, the BFR+BRJ group maintained a greater increase in the relative average torque of the left and right legs in the range of 51–100 repetitions compared to the pre-intervention period. Thus, both BFR and BFR combined with BRJ intervention were able to improve knee extensor force output during prolonged contractions compared to pre-intervention, with the BFR+BRJ group showing a more significant advantage in the second half of the contraction. This suggests that BFR combined with BRJ intervention may better improve the fatigue resistance of the knee extensors during prolonged contractions.

### Pre- and Post-intervention comparisons of peak torque, peak power, and average power between fatigued and non-fatigued conditions

**FIG. 4:**
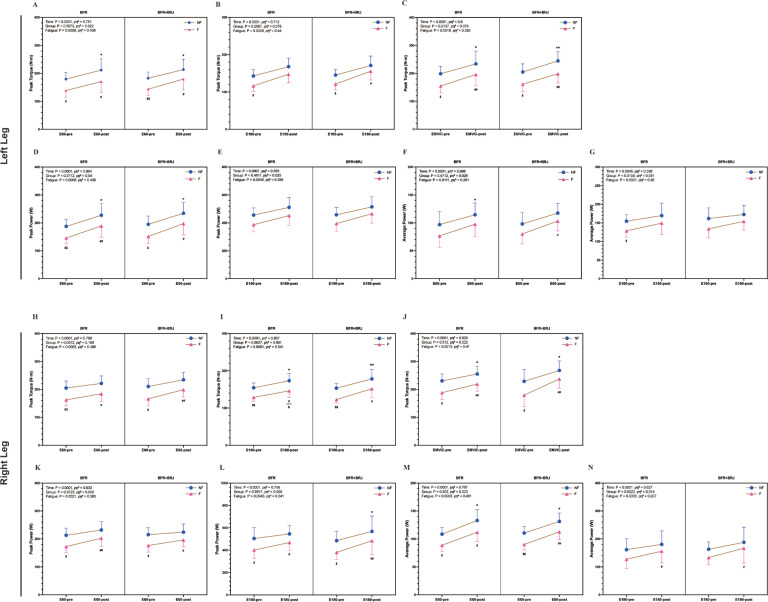
Knee Extensor Performance: Peak Torque, Average Power, and Total Work across Fatigued and Non-Fatigued States (pre- and post-Intervention). Note: E60, extensors at 60°/s; E180, extensors at 180°/s; EMVIC, extensors maximum voluntary isometric contraction. Significant differences between NF-pre and NF-post are denoted by *, and between F-pre and F-post by #. Differences between pre-NF and pre-F are represented by $, while those between post-NF and post-F are represented by &. Statistical analysis was conducted using a three-way repeated-measures ANOVA (group × fatigue × time) with Bonferroni’s multiple comparisons test for post hoc analysis. Main effects for time (pre vs. post) and fatigue (NF vs. F) were statistically significant, while no significant interaction effects (group, group × time, group × fatigue, time × fatigue, or group × fatigue × time) were detected. Statistical significance levels are: * P < 0.05, ** P < 0.01, and *** P < 0.001.

The performance of the knee extensor muscles under fatigued and non-fatigued conditions is shown in Figure 4. At pre-intervention, the left leg showed significantly lower peak torque in the fatigued state compared to the non-fatigued state at 60°/s (P < 0.05, Figure 4A), 180°/s (P < 0.01, Figure 4B), and MVIC (P < 0.05, Figure 4C). A similar trend was observed in the right leg at 60°/s (P < 0.05, Figure 4H), 180°/s (P < 0.01, Figure 4I), and MVIC (P < 0.05, Figure 4J). At post-intervention, peak torque in the non-fatigued state significantly increased in both groups (60°/s, 180°/s, and MVIC: P < 0.05, Figures 4A-C, Figures 4H-J). In the fatigued state, the BFR+BRJ group showed greater improvements than the BFR group (60°/s: P < 0.01, Figure 4H; 180°/s: P < 0.05, Figure 4B). In the right leg at 180°/s, post-intervention-fatigued peak torque remained lower than post-intervention-non-fatigued, but showed significant improvement compared to pre-intervention-fatigued in the BFR+BRJ group (Figure 4I).

For peak power, at pre-intervention-fatigued, the left leg showed significant reductions at 60°/s (P < 0.05, Figure 4D), while the right leg showed reductions at 60°/s (P < 0.05, Figure 4K) and 180°/s (P < 0.01, Figure 4L) compared to the non-fatigued state. At postintervention, peak power in the non-fatigued state significantly improved in both groups (P < 0.05, Figures 4D, K). The BFR+BRJ group also showed significant improvement in the right leg at 180°/s (P < 0.05, Figure 4L). In the fatigued state, peak power increased in both groups (60°/s: P < 0.01, Figures 4D, K; 180°/s: P < 0.05, Figure 4L).

For average power, at pre-intervention-fatigued, the right leg showed significant reductions at 60°/s (P < 0.05, Figure 4M), while the left leg showed reductions at 180°/s (P < 0.05, Figure 4G) compared to the non-fatigued state. At post-intervention, average power in the nonfatigued state significantly increased in both groups (P < 0.05, Figures 4F, M). In the fatigued state, the BFR group showed moderate improvements (P < 0.05, Figures 4M, N), while the BFR+BRJ group exhibited more pronounced enhancements, particularly in the right leg at 60°/s (P < 0.01, Figure 4M). Both BFR and BFR+BRJ interventions improved knee extensor performance, especially in the fatigued state. The BFR+BRJ group showed greater improvements, with significant advantages in peak power and torque.

### Changes in the rate of decline in peak torque, peak power, and average power of fatigued knee extensors pre- and post-intervention

**FIG. 5:**
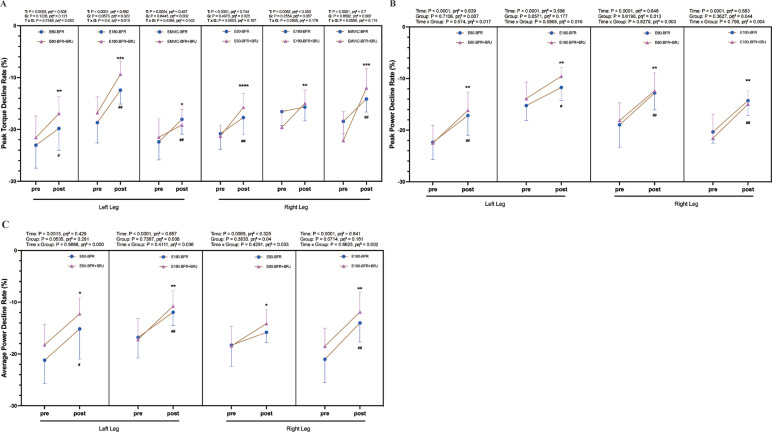
Pre- and Post-Intervention Changes in Decline Rate of Peak Knee Extensor Torque, Peak Power, and Average Power. Note: Values are presented as mean ± standard deviation (SD). Significant differences between pre- and post-intervention are indicated by # for the BFR group and * for the BFR + BRJ group (* P < 0.05, ** P < 0.01 and *** P < 0.001).

Figure 5 illustrates the pre- and post-intervention changes in the decline rates of peak knee extensor torque, peak power, and average power under fatigue. After four weeks of intervention, the BFR group and the BFR+BRJ group demonstrated significant reductions in the decline rate of peak knee extensor torque in both legs under 60°/s and MVIC conditions (P < 0.05), with the BFR+BRJ group showing a more pronounced reduction in the right leg (P < 0.01, Figure 5A). At 180°/s, although the right leg of the BFR group did not show a significant reduction, the left leg of the BFR group (P < 0.01) and both legs of the BFR+BRJ group (P < 0.01) exhibited significant reductions. Notably, in the BFR+BRJ group, the improvements in both legs were significantly greater than in the BFR group, with the right leg showing particularly prominent improvements.

For peak power, both the BFR group and the BFR+BRJ group demonstrated significant reductions in the decline rates for both legs at 60°/s and 180°/s compared to baseline (P < 0.05, Figure 5B). Regarding average power, while the right leg of the BFR group at 60°/s did not show significant reductions, significant declines were observed in the BFR group (left leg at 60°/s and 180°/s; right leg at 180°/s) and in the BFR+BRJ group (both legs at 60°/s and 180°/s) (P < 0.05, Figure 5C). Compared to the BFR group, the BFR+BRJ group showed superior advantages in mitigating the decline in knee extensor strength under fatigue.

### Anaerobic power test between pre- and post-intervention

**FIG. 6:**
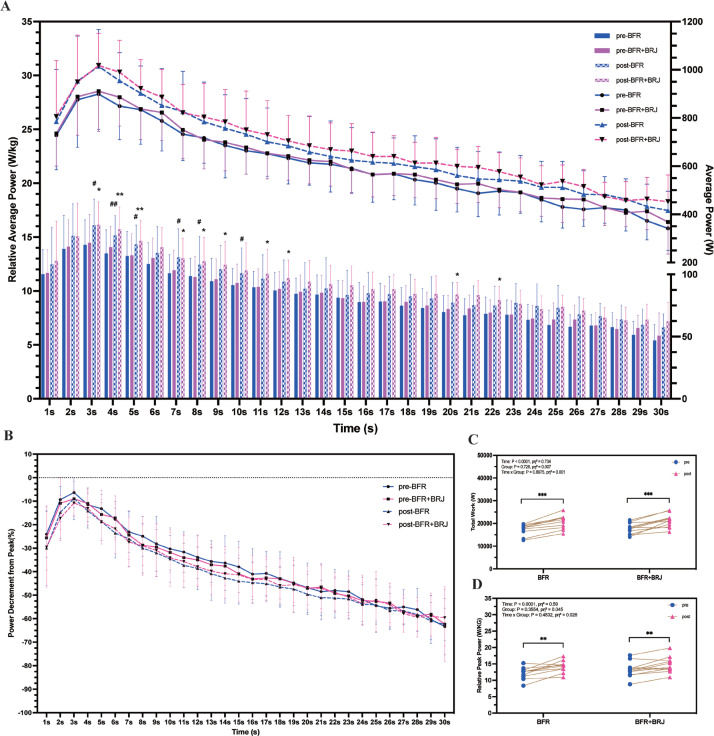
Changes in 30-Second Anaerobic Power Test Indicators Pre- and Post-Intervention. Note: Values are presented as mean ± standard deviation (SD). Significant differences between pre- and post-intervention in Figure A are indicated by # for the BFR group and * for the BFR + BRJ group (* P < 0.05, ** P < 0.01, and ***P < 0.001).

The average power per unit of time was recorded in full throughout the test (Figure 6A). Compared to pre-intervention, the BFR intervention resulted in a significant increase in relative average power in the first 10 seconds (P < 0.05), while the BFR+BRJ intervention extended this effect to 12 seconds, with significant increases at 20 and 22 seconds (P < 0.05). Figure 6B shows the rate of decrease in average power per unit time relative to peak power. Total work (Figure 6C, P < 0.001) and relative peak power (Figure 6D, P < 0.01) were significantly higher in both the BFR and BFR+BRJ groups after the intervention compared to the pre-intervention.

## DISCUSSION

This study verified the effective role of BFR intervention and BFR combined with BRJ intervention in improving the maximal strength of the knee flexor and extensor muscles. Specifically, in the 60°/s test, which is used to assess the maximal strength of the muscle, both interventions significantly improved the peak torque of the knee extensors and flexors of both legs, further supporting the effectiveness of BFR in improving muscle strength [12]. However, in the 180°/s and MVIC tests, which assess power characteristics and isometric maximal strength, the BFR+BRJ group showed superior effects on peak torque, peak power, and average power. This may be attributed to elevated plasma nitrite levels induced by BRJ supplementation, which enhances NO synthesis. Research involving both humans [36] and rodents [20] substantiates the notion that increased NO levels, resulting from dietary nitrate consumption, can improve the contraction velocity and strength of fast-twitch muscle fibers by influencing excitation-contraction coupling at the Ca^2+^ release level. Nonetheless, BRJ supplementation does not entirely elucidate this phenomenon, as the time to peak torque was not significantly diminished across all assessments. Furthermore, NO may slightly inhibit isometric force generation [37] and the beneficial effect of nitric oxide on neuromuscular function in combination with BFR remains to be elucidated. Consequently, the combination of BFR and BRJ may exhibit complimentary or inhibitory effects that require additional validation.

The BFR intervention exhibited good results in slowing down the loss in torque during prolonged contractions of the knee extensors, significantly enhancing the torque during the 100 repeat isometric tests. Despite variations in the test protocol, Apiwan et al. reported analogous results, indicating that five weeks of knee extensor BFR training significantly enhanced the area under the 30-second force curve (MVIC30) and repetitions at 20% of onerepetition maximum (1RM) [38], thereby suggesting the beneficial effects of BFR on knee extensor fatigue resistance. The BFR+BRJ group had enhanced torque maintenance, showing a more significant advantage in the second half of the contraction. The BFR+BRJ group also showed a greater power output for a longer period in the 30-second anaerobic power test, therefore supporting the effect of BRJ in extending high-intensity power output during anaerobic exercise. As previously mentioned, BRJ can increase blood flow by means of NO, thereby improving the supply of energy and enabling the lactate clearance. Recent studies suggest that skeletal muscle may function as a primary site for nitrate storage and metabolism [25]. During physical activity, the higher oxygen demand may facilitate the fast conversion of nitrate stored in skeletal muscle to nitrite through the nitrate reductase route, hence increasing NO synthesis [39]. Nevertheless, the synergistic action of BRJ and BFR may extend beyond the promotion of blood circulation. NO-induced vasodilation may amplify metabolic responses by enhancing reperfusion after BFR-induced hypoxia. This may improve oxygen use and energy production rates in muscle cells by regulating mitochondrial respiratory chain complexes and mitochondrial biogenesis in skeletal muscle [19, 36]. Furthermore, hypoxic circumstances have been demonstrated to promote the conversion of nitrite to NO, which may enhance nitrate utilization and NO production in skeletal muscle during BFR, hence eliciting a more pronounced metabolic response [24, 25]. Our findings substantiate the efficacy of BFR in conjunction with BRJ in postponing muscle fatigue and sustaining elevated muscle performance over prolonged durations.

Recovery from fatigue is a crucial indicator for evaluating fatigue resistance. Our investigation revealed that both BFR intervention alone and BFR in conjunction with BRJ intervention significantly reduced the decline in peak torque, average power, and total work during 60°/s, 180°/s, and MVIC assessments post-fatigued. Although BFR intervention alone has demonstrated the ability to improve post-exercise performance recovery [40], it is noteworthy that the BFR combined with BRJ intervention appeared to yield more pronounced improvements in the tests. On the one hand, BRJ been demonstrated to possess significant antioxidant and anti-inflammatory effects, which appear to effectively clear the substantial ROS produced in skeletal muscle during intense exercise. This reduces the inflammatory response caused by micro-damage to muscle fibers [17], thereby delaying exercise-induced fatigue and accelerating post-exercise recovery [18]. On the other hand, the local hypoxia induced by BFR increases the oxygen demand of muscles, stimulating the upregulation of hypoxia-inducible factor-1α (HIF-1α), which in turn enhances capillary formation and muscle metabolic adaptability [7]. Concurrently, BRJ facilitates vasodilation by elevating NO levels, thereby mitigating hypoxic stress, and further advancing microcirculation remodeling, which continuously enhances muscle adaptability following fatigue [41]. This circulation-boosting effect not only accelerates the supply of oxygen and nutrients after fatigue, but also enhances muscle recovery and adaptation to subsequent exercise by boosting angiogenesis and mitochondrial function.

The results of this study could provide a theoretical basis for the use of BFR and BRJ to optimize the training methods of athletes and improving the efficiency of sports recovery. The present study exhibited some limitations: Firstly, the study population was confined to male students majoring in physical education, hence constraining the generalizability of the findings; additionally, the sample size should be augmented in future research to encompass diverse genders and age groups. Secondly, the long-term effects of BFR+BRJ and the sustainability of short-term interventions were not evaluated, necessitating additional long-term follow-up research. Ultimately, the lack of systematic and standardized monitoring of participants’ habitual dietary patterns and caloric intake represents a limitation of this study. It is necessary to consider the potential confounding effects of dietary changes on the results.

## CONCLUSIONS

BFR combined with BRJ is an effective training method for improving muscle strength performance and fatigue resistance. Based on the findings of this study, the following evidence-based recommendations are proposed for sports training implementation:

1. Low-load blood flow restriction (BFR) training (30% maximum force) combined with moderate-dose nitrate supplementation (8 mmol/day via BRJ) constitutes a synergistic strategy to effectively enhance skeletal muscle force and fatigue resistance.
2. It is recommended that competitive athletes and fitness enthusiasts with comparable training backgrounds should integrate this combined intervention with traditional resistance training regimens. Particularly for sports that require both muscular strength and endurance (e.g., Soccer, basketball, volleyball, wrestling, rowing, middle distance running).
